# A novel hardmask-to-substrate pattern transfer method for creating 3D, multi-level, hierarchical, high aspect-ratio structures for applications in microfluidics and cooling technologies

**DOI:** 10.1038/s41598-022-16281-5

**Published:** 2022-07-16

**Authors:** Sougata Hazra, Chi Zhang, Qianying Wu, Mehdi Asheghi, Kenneth Goodson, Ercan M. Dede, James Palko, Sreekant Narumanchi

**Affiliations:** 1 Department of Mechanical Engineering, Stanford University, Stanford, USA; 2 Electronics Research Department, Toyota Research Institute of North America, Ann Arbor, MI USA; 3 Department of Mechanical Engineering, University of California-Merced, Merced, CA USA; 4 National Renewable Energy Laboratory, Golden, CO USA

**Keywords:** Mechanical engineering, Techniques and instrumentation, Nanoscale devices, Nanoscale materials, Techniques and instrumentation, Applied physics

## Abstract

This letter solves a major hurdle that mars photolithography-based fabrication of micro-mesoscale structures in silicon. Conventional photolithography is usually performed on smooth, flat wafer surfaces to lay a 2D design and subsequently etch it to create single-level features. It is, however, unable to process non-flat surfaces or already etched wafers and create more than one level in the structure. In this study, we have described a novel cleanroom-based process flow that allows for easy creation of such multi-level, hierarchical 3D structures in a substrate. This is achieved by introducing an ultra-thin sacrificial silicon dioxide hardmask layer on the substrate which is first 3D patterned via multiple rounds of lithography. This 3D pattern is then scaled vertically by a factor of 200–300 and transferred to the substrate underneath via a single shot deep etching step. The proposed method is also easily characterizable—using features of different topographies and dimensions, the etch rates and selectivities were quantified; this characterization information was later used while fabricating specific target structures. Furthermore, this study comprehensively compares the novel pattern transfer technique to already existing methods of creating multi-level structures, like grayscale lithography and chip stacking. The proposed process was found to be cheaper, faster, and easier to standardize compared to other methods—this made the overall process more reliable and repeatable. We hope it will encourage more research into hybrid structures that hold the key to dramatic performance improvements in several micro-mesoscale devices.

## Introduction

**Figure 1:**
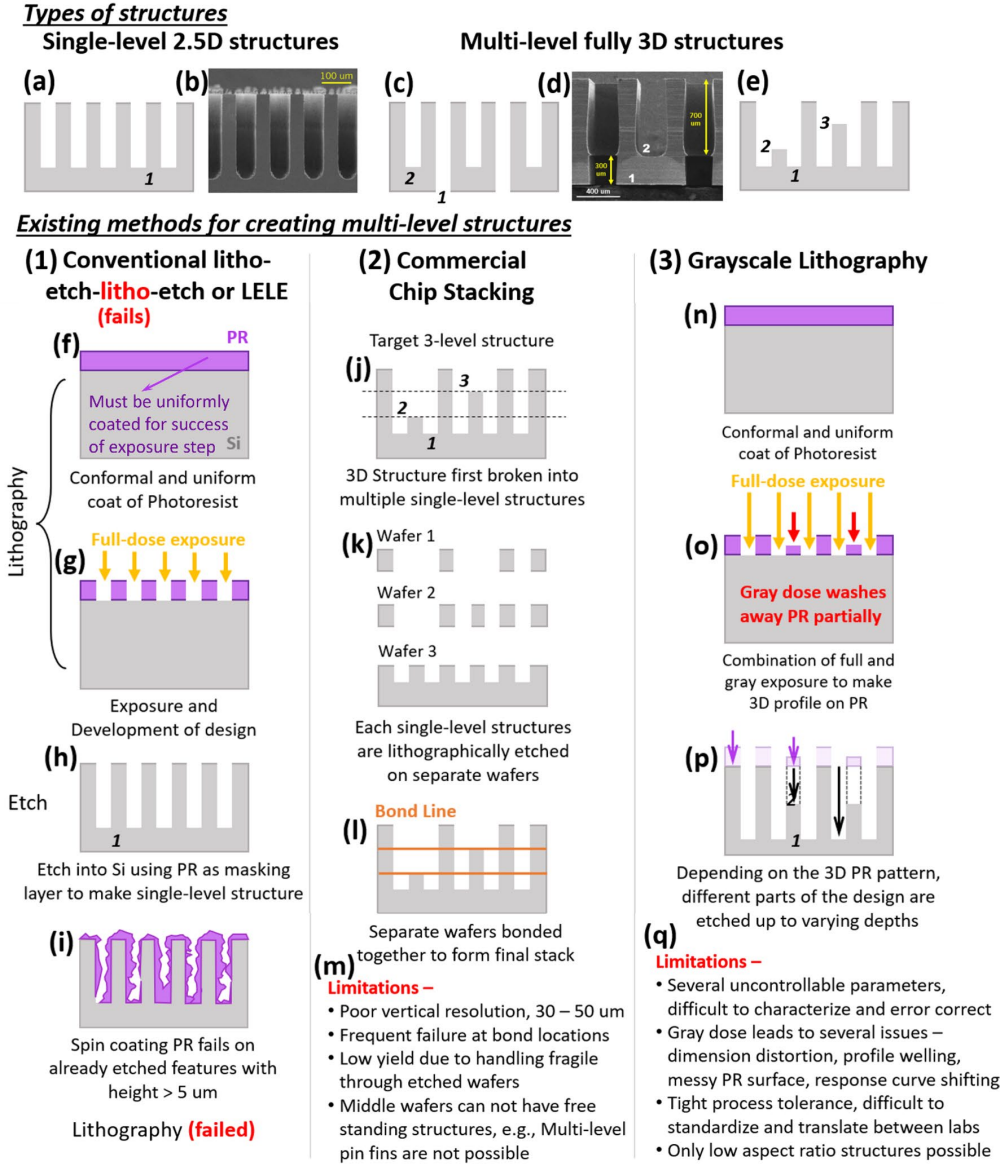
Two types of structures, 2.5D and 3D*.* (**a**,**b**) Shows single-level structures, where all the features are of the same depth/height. These are also the structures that we will refer to as 2.5D structures throughout the rest of this letter. (**c**–**e**) In contrast to 2.5D structures, we show two different fully 3D structures which are multi-level; different parts of the wafer have different etch depth/height. (**d**) Is fabricated version of (**c**). (1) Conventional LELE (**f**) Spin coat PR, the uniformity of the PR layer is critical for the success of the downstream processes (**g**) Full exposure and development to make a 2D pattern on the PR (**h**) Deep Si etch to make a single-level structure first. (**i**) Second round of PR spin coating fails if etched structure height is taller than PR layer thickness (4–10 μm). (2) Chip Stacking (**j**) Target 3-level structure. (**k**,**l**) Multi-level structure first broken into multiple single-level structure which need to be made on separate wafers and then bonded together. (**m**) Limitations of chip stacking. (3) Grayscale Lithography (**n**,**o**) After PR coating, combinations of full dose (energy) and partial dose lithography is performed to create a 3D structure in the PR. (**p**) Etching transfers the 3D pattern from the PR to the Si underneath. (**q**) Limitations of grayscale lithography primarily associated with partial gray doses.

Advances in lithography based micro-nano processing techniques have revolutionized the technology around the world for its ability to cost effectively mass produce structures ranging from sub-10 nm lengthscale all the way up to millimeter scale. Some of these structures include nanometer scale electronics components like FETs, IGBTs^1^, sub-micron features like optical waveguides^2^, Fresnel lenses^3^, photonic devices^4^, and micro-nanofluidic devices^5^. Slightly larger micro (1–100 μm) and meso (0.1–1 mm) scale features are even more useful in modern technology and has seen myriads of applications in microfluidics^6^, cooling technologies^7,8^, battery research^9^, sorption–desorption^10^, desalination^11^ and catalysis^12^. Although ubiquitous, versatile, and indispensable as a micro-nano manufacturing technique, conventional cleanroom-based lithography suffers from one major limitation. This type of processing can efficiently create only 2.5D or single-level structures (Fig. 1a,b) but is unable to reliably create multi-level, hybrid, 3D hierarchical structures (structures with more than one level of height or depth as shown in Fig. 1c–e) of depths more than 1–5 μm. Through conventional LELE (Litho-Etch Litho-Etch) route, a 2D design/pattern (full control available over the feature design in 2D) is first lithographically laid on a sacrificial mask layer [usually, a photosensitive polymer called photoresist (PR)] on the wafer. This mask is now used as protection to etch the exposed part of the design onto the wafer. Through one round of ‘lithography + etching’ the entire design can be etched to only one specific depth thus giving rise to a single level structure. Conventional LELE cleanroom process would normally require multiple rounds of back-to-back ‘lithography + etching’ to achieve the desired multi-level structures (Fig. 1f–i). The bottleneck arises due to unsatisfactory second round of lithography (Fig. 1i) on wafers which have already gone through one round of ‘lithography + etching’ and thus have etched features (height ≥ 5 μm) in them. This comes as a major manufacturing hurdle in a time when hybrid structures hold the key to dramatic improvements in the performance of existing devices. (Additional details on usefulness of hybrid structures can be found in the “Impact” section.)

Multi-level, 3D structures can be made with ease from soft materials like PDMS (polydimethylsiloxane), thermoplastics using deforming techniques (two-step soft lithography^13^, sequential thermal^14^ and UV^15^ Nano Imprint Lithography (NIL), Capillary Force Lithography^16^, Nano Transfer Printing (NTP)^17^) but reliable methods for fabrication of 3D multi-level structures in rigid material like silicon is still lacking^18^. Recently, two-photon lithography has enabled the fabrication of complicated fully 3D patterns in photopolymers^19,20^, but these systems have an extremely small print volume (Nanoscribe GT, a state-of-the-art multi-photon system used in academia and industry can print a maximum volume of 300 × 300 × 300 μm^3^) with equally long write times of more than 12 h per structure. This reason makes two-photon lithography prohibitively expensive to use and difficult to integrate in commercial mass manufacturing scenarios^20–22^. Another technique called grayscale lithography^23–44^ has gained some traction in recent years, although, this method is often expensive, tedious, difficult to characterize^22,45–48^. In this approach, several gray doses which have energy less than the full dose-to-clear energy are used to illuminate the photoresist (PR). The PR in this gray dose exposed zones undergo partial photochemical reaction, and when developed, only some part of the resist gets washed away—precisely controlling the energy and focal plane of the exposing light results in a multi-height 3D structure in the resist and subsequently transferred to the substrate underneath (Fig. 1n–q). However, it was quickly found that the gray exposure doses were associated with several uncontrollable problems^22,45–49^. Morgan et al. attested to this difficulty by citing the lack of standardization of grayscale lithography process steps. According to them, this arises because of severely limited control over several parameters that is inherently associated with gray dose exposure^47^. Some of these challenges encountered in grayscale lithography are—complicated and expensive mask modelling^22,48^, dose dependent dimension distortion which gets worse at sub-10 μm feature sizes^49^, feature size dependent shifting of characteristic response curve of photoresists^49^, profile welling and sidewall tapering at gray doses, messy post-development PR surface. All these problems make the target resist profile extremely difficult to achieve^22^. These issues must be perfectly addressed through extensive experimental characterization and tedious numerical model-based error correction for overall success of the process^22^. Morgan et al. further goes on to states that making accurately controlled gray features is so heavily dependent on process conditions and operate within such tight process tolerances that transferring recipe or process knowledge from one lab to another is almost impossible. Small changes in process equipment and environment causes drastic changes in the grayscale process^47^.

Recently, an ingenious double-sided processing technique has been developed by several researchers^50–53^ and using this technique they were able to create 2-level 3D manifold structures for high power electronics hot-spot (25–100 mm^2^ footprint) cooling. Later, Hazra et al. successfully demonstrated scalability of this process flow to create extremely large area (≥ 500 mm^2^) high heat flux 3D manifolded micro-coolers^54^. However, this method is only suitable for creation of very specific 2-level structures which can be made via intersection of two designs etched from both sides of the wafer. Furthermore, the yield of 3D structures made via conventional or double-sided micro-lithography techniques on rigid Si wafers, drop drastically to about 50% because of manual handling of fragile wafers which have already gone through a round of deep Si etch^54,55^. Thus, commercially, the creation of taller (≥ 10 μm) multi-level structures have traditionally been performed via chip stacking methods^50,56^. In this approach, a fully 3D design is split into several different 2.5D structures; these 2.5D structures are fabricated in separate wafers using conventional ‘lithography + etching’ which are then stacked together using solder die-attach or thin eutectic bonding technologies (Fig. 1j–m). The wafer thicknesses used for each of these separate layers determine the step heights achievable through this process and often, to achieve small step heights, the wafers need to be thinned down using a back-grinding tool. Wafer thinning is not possible below 30–50 μm which puts quite a large limitation on the minimum step height or vertical resolution of this process. Moreover, extremely thin wafers are prone to warping, chipping, and breakage. The final bonded chip-stacked configurations are short-lived and unreliable, the bonding sites being the primary source of failure. These issues present themselves more frequently in devices that go through massive cyclic thermal or mechanical stresses^23,24^, especially, in high heat flux microfluidic cooling devices. Also, chip stacking techniques have their limitations in terms of the device configurations it can fabricate, since the middle wafer layers of the stack cannot have free standing structures (for e.g., multi-level pin fin array structures cannot be made using this technique). Thus, current microfabrication community is in desperate need of a standardized, easily characterized process to make high aspect ratio, tall (≥ 100 μm) micro-mesoscale multi-level structures that is simple, cost-effective, can operate between reasonable process tolerances, and thus ultimately easily translatable from one lab to another.

In this paper, we have described a novel silicon dioxide to silicon pattern transfer process which can reliably create multi-level structures using photolithography techniques and simultaneously solve several of the practical challenges that arise while employing existing state-of-the-art methods like chip stacking and grayscale lithography. The pattern transfer process is achieved through a single shot deep silicon etching step which translates into an improvement in manufacturing yield by more than 40%. Furthermore, Si:SiO_2_ etch selectivity is more than an order of magnitude higher compared to Si:PR etch selectivities^25–30,49^, thus enabling us to create really tall (up to 500 μm), high aspect ratio (~ 10–15) structure the likes of which will be immensely useful in applications that rely on mesoscale features. The process described employs full-dose exposure and thus circumvents all the challenges and difficulties associated with partial dose gray exposure. Removing the gray exposure step simultaneously eliminates the “hard-to-control” parameters that are inherently associated with partial exposure steps in gray-lithography^47^. The only parameters to be characterized are associated with etching silicon dioxide and silicon, thus making this novel process easy to generalize, and not require extremely tight process tolerances. This letter mentions a simple characterization method and details data on SiO_2_ and Si etch specific to the tools and step conditions used. Coupled with easy characterization and standardizability, the process also employs very commonly used cleanroom-based tools and processes to create multi-level microstructure—this makes knowledge transfer from one lab to another much easier. Finally, this letter shows proof-of-concept of this method via performing two rounds of ‘lithography + etch’ and shows SEM images of several 2-level and 3-level microstructures made. However, the possibilities in terms of structure types, topologies, configurations, and length-scale are endless. Finally, this letter ends by listing some exciting applications of these novel hybrid structures whose fabrication is now made possible and which could pave the way for the next generations of high-performance microfluidics and cooling technologies.

## Methods

The novel process described in this paper draws inspiration from multi-lithography LELE techniques and grayscale lithography. It cleverly combines the two for reliable fabrication of multi-level 3D structures and simultaneously solve several practical challenges associated with PR-based grayscale lithography technique. In conventional lithography (Fig. 1f–h), creation of etched features usually follows these steps—coating Photoresist (PR) on the wafer (Fig. 1f); exposing 2D design on the PR with light of appropriate wavelength and energy which causes a photochemical reaction in the PR and makes it dissolvable in a special solvent called the developer (developers are usually highly dilute solutions of extremely corrosive bases like TMAH, Tetramethylammonium hydroxide). After exposure, the developer is used to wash away the parts of the PR that had been exposed in the previous step, thus leaving behind a 2D pattern of PR on the wafer surface (Fig. 1g). Deep Reactive Ion based Bosch Silicon etch is subsequently performed to etch away the exposed parts of the wafer up to a desired depth and we are left with a single-level structure with all the features having the same depth into the silicon wafer (Fig. 1h). To achieve a multi-depth structure using this conventional technique, a process flow called LELE (litho-etch-litho-etch) is used. In this process, the sequence of steps ‘lithography + etch’ needs to be repeated multiple times with a different exposure design and different etch times in each step. The primary challenge arises in the second lithography step where PR is attempted to be spun on the wafer with features already etched in it. The spin coating process works via PR being puddle dispensed at the center of a Silicon wafer spinning at a high RPM, making it spread radially outward to create a thin, uniform, and conformal coating over the wafer. The spinning process on an already etched wafer is satisfactory (thin and uniform) when the PR thickness (4–10 μm) is much larger compared to the etch height of the features. Thus, in some cases of IC fabrication, where the already etched feature height is ≤ 1–4 μm, the LELE process works perfectly. However, in several useful applications of microfluidics, liquid cooling, optics and semiconductor fabrication, these etch depths are of the micro-meso scale and can range anywhere from 10 μm to 500–600 μm. PR spinning on larger step heights (more than 5–10 μm) lead to unsatisfactory coating (Fig. 1i). Several problems like streaking (PR layer being wrinkled after hitting an etched feature or obstacle), fingering (PR getting trapped in a deep cavity/channel and progressing along those channels only), and incomplete coverage (PR hitting the corner of an etched feature and failing to cover the rest of the wafer) mar the spin coating process in the second rounds of lithography. This causes failure of the downstream exposure process, whose success relies exclusively on the uniformity of the PR coat—thus leading to failure of the overall process.

**Figure 2:**
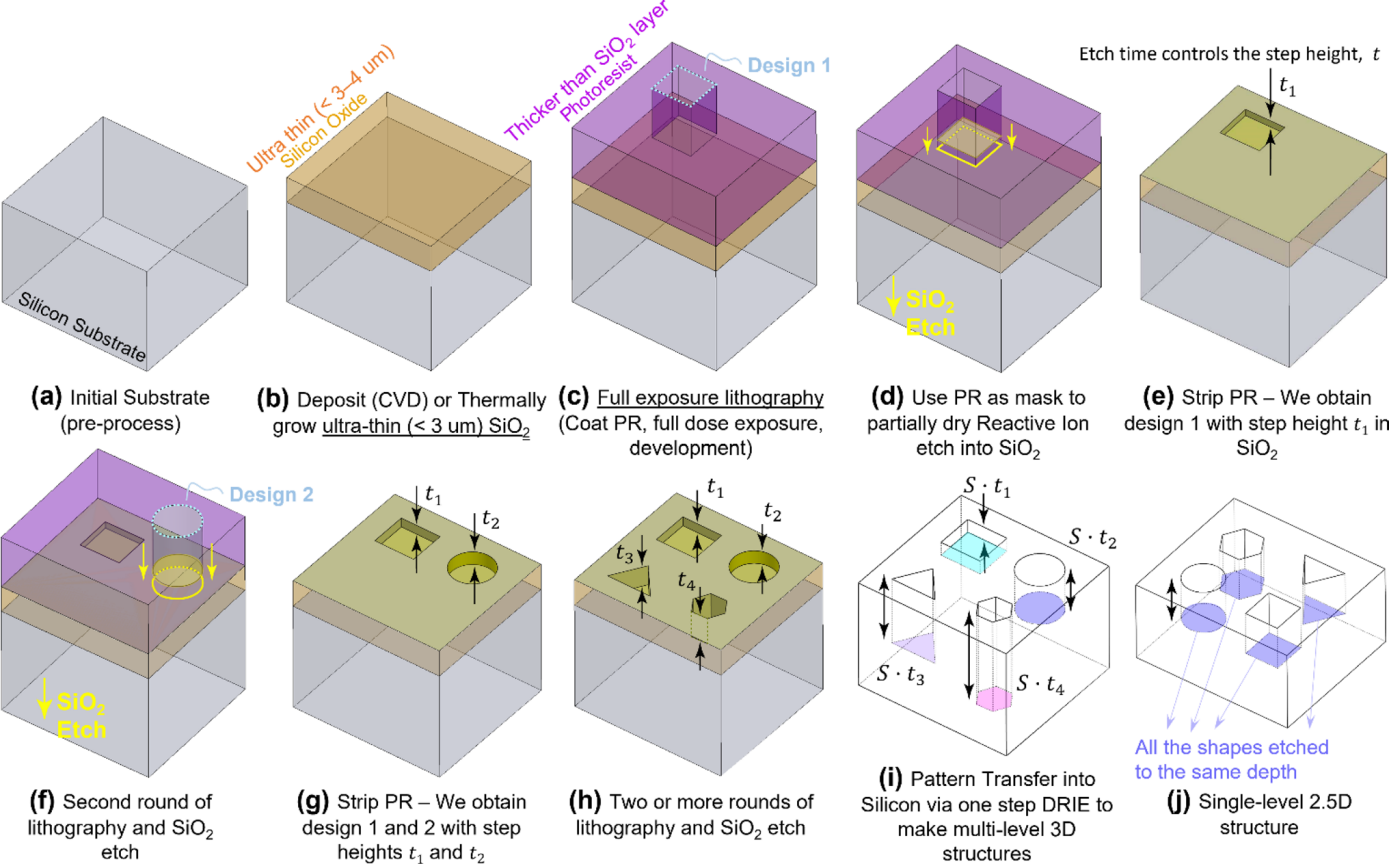
Process flow for creation of multi-level structures using novel approach. (**a**) Clean bare wafer without features; (**b**) Intermediate ultra-thin masking material is deposited—in our case, SiO_2_ is CVD deposited; (**c**) Photoresist (PR) spinning is uniform, this process is not hindered because it is thicker than the underlying SiO_2_ layer, exposure of Design 1, and development; (**d**) Using PR as mask layer, the underlying SiO_2_ is etched to a precise amount, ${t}_{1}$; (**e**) Stripping PR; (**f**) Second round of lithography is performed—in this situation PR thickness is at least 1.5 times the maximum SiO_2_ feature thickness already on the wafer, thus spin coating process is successful, yielding a thin conformal coat all over the 3D featured SiO_2_. This time design 2 was etched in SiO_2_ to a different depth, ${t}_{2}$; (**g**) After two rounds of litho, a 2-level structure is made on the SiO_2_; (**h**) After two more rounds of litho, 2 more levels can be made. In theory, $n$ rounds of lithography is able to make at least $n$ levels in the structure; (**i**) The wafer with a 3D structured SiO_2_ layer is now etched in a deep Si Reactive Ion Etcher (RIE) to vertically scale the SiO_2_ 3D pattern by the Si:SiO_2_ selectivity (which is around 200–300 for our case) and transfer to the silicon wafer underneath. Finally, we are left with an $n$ level, high aspect ratio structure, deep structure in Si; (**j**) In contrast to the multi-level structure, this is a single-level structure shown for comparison.

We identified two major problems in conventional methods that lead to complicacy in reliable fabrication—unsatisfactory PR coating issues on etched structures with height ≥ 5 μm in LELE processing; and unavoidable and uncontrollable issues associated with partial gray dose exposure in grayscale lithography. In this context, we have invented a novel process flow using commonly used cleanroom tools which mitigates all these problems and enables us to create multi-level hierarchical structures with ease. Inspired by grayscale lithography’s principle of 3D patterning the etch mask material, we have first introduced an ultra-thin, sacrificial layer of SiO_2_ in between the PR and the Silicon wafer; the SiO_2_ layer now acting as the masking material during the deep Si etching process instead of PR. The idea is to perform multiple rounds of conventional LELE lithography to pattern this newly introduced SiO_2_ mask layer, instead of attempting to directly pattern the Silicon underneath. After this, by deep Si etching, this 3D multi-level pattern in the SiO_2_ gets scaled vertically and transferred to the silicon—the overall process flow is shown schematically in Fig. 2. In the first step of this process flow, the thin (1–3 μm) layer of SiO_2_ is first deposited on the wafer through Chemical Vapor Deposition (CVD) at 250–350 °C or thermal oxidation process (≥ 850 °C) (Fig. 2b). Alternatively, to make the process more BEOL (back-end-of-line) friendly, high density plasma enhanced CVD (HDPECVD) process can be used which can deposit high quality SiO_2_ but at a much lower temperature of 90–120 °C by using directional plasma to enhance the deposition process. Following the SiO_2_ layer growth/deposition, multiple rounds of lithography is performed on the SiO_2_ layer with different design and SiO_2_ etch time in each of the rounds (Fig. 2c–h). Since the SiO_2_ layer is ultra-thin, the maximum etch step heights (≤ 3 μm) in SiO_2_ is always less than the PR layer thickness (4–10 μm) spreading over them during the spinning process. These low aspect ratio features in the SiO_2_ do not interfere with the PR spinning process, thus giving rise to perfectly uniform and conformal PR coats on the SiO_2_ layer during the multiple lithography steps (Fig. 2f). After the desired 3D profile has been etched in the SiO_2_ layer through multiple lithography rounds, the wafer is placed in a deep Si etcher which achieves anisotropic profiles in Si via a time-multiplexed deep reactive ion (DRI) process often also termed Bosch etching process^54^. This etching step is a one-shot process which scales the 3D profile in the SiO_2_ vertically by the Si:SiO_2_ etch selectivity and transfers it to the Silicon underneath (Fig. 2i). Since this etching step is a single-shot process, it also eliminates manual handling of fragile deep etched wafers like in chip stacking or conventional LELE process, thus improving process yield from 50 to 90%. Furthermore, these structures are now monolithic or made of a single bulk Si substrate, which eliminates the several bonding layers that would be required in chip stacking approach—this increases device reliability and robustness; the thermal and mechanical stress cycling induced failures in chip stacked configurations are effectively avoided. All the aforementioned effects combined would result faster processing time, more yield, higher throughput in industrial mass manufacturing scenarios and ultimately cheaper devices. The two major issues mentioned at the beginning of this section are also mitigated through this process flow—the use of ultra-thin SiO_2_ eliminates the PR spin coating issues over etched steps in SiO_2_, and performing full dose exposure-based lithography to pattern the SiO_2_ layer gets rid of the difficult to characterize problems associated with gray dose lithography. Moreover, SiO_2_ as a etch hard-mask provides very high selectivity of etch (200–300) with respect to Si, which is more than twice that of maximum Si:PR selectivity of 80–100. This enables us to easily make meso-scale structures taller than 500 μm using extremely thin (≤ 3 μm) SiO_2_.

It is also important to note that all the steps used to make up this process flow like lithography (spinning PR, exposing sub-10 μm feature design, development), SiO_2_ deposition or growth on Si wafer, SiO_2_ reactive ion etch (RIE) and DRI etch of silicon are very commonly employed in the cleanroom microfabrication community. This enables easy transfer of process knowledge from one lab to another, something that is almost impossible for grayscale technology^47^. Furthermore, this process flow uses only full exposure lithography, which has been extensively characterized and documented for different types and thicknesses of positive and negative resist. The elimination of gray dose exposure deals away with some of the unavoidable issues associated with grayscale technology like gray dose induced PR response curve shifting, gray dose profile welling, messy surface post-development, gray dose dependent dimension distortion^22,45–49^ etc. In absence of these issues, expensive and tedious experimental and numerical profile error correction steps that would otherwise be required, are also effectively avoided. The only characterization required is related to the etch of SiO_2_ and Si, both of which have also been extensively characterized by numerous previous researchers. Despite these processes being very common and their characterization data widely available in the microfabrication community, we have detailed some characterization data later in the section specific to the tools and recipes we have employed to provide a starting point for anyone looking to fabricate such structures. Information about the specific tools and recipes used in our study can be found in the Supplementary Information (SI) Table 1. The SI Table 3 also contains a tabular comparison of this novel method with the existing processes of chip stacking and grayscale lithography (masked and maskless).

Preliminary tests using this novel process flow (Fig. 2) have demonstrated the ability to create 3D hierarchical features of nominal dimensions (width) ~ 5–10 μm with aspect ratios (height to width ratio) as large as 10–15. The resolution can be further improved to sub-500 nm scale by using e-beam lithography instead of conventional photolithography. The process flow of creating multi-level structures has been tested 5 times with different magnitudes of step heights (250 nm through 1.5 μm) to establish reliability and repeatability.

**Figure 3:**
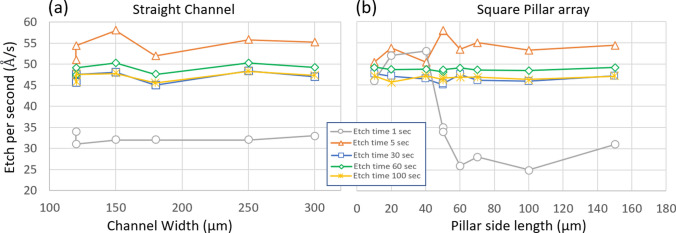
To establish repeatability and standardizability of our method, precise characterization of the oxide etch rate is imperative. The characterization wafer consisting of straight channels and square pillar arrays were etched for varying durations (1–100 s) and the etch per second in Å/s has been plotted as a function of the feature type and dimensions. The raw data used to plot these curves can be found in the Supplementary Information. Etch per second was found to be stable around 5 nm/s. However, the important observation is the fact that no Aspect Ratio Dependent Etching (ARDE) in SiO_2_ is noticed (the maximum variation in etch per second in different geometries was found to be ~ 1 nm/s) showing a consistent etch per second value for different feature dimension and loading conditions. Etch per second was seen to be more influenced by total etch time, especially when total etch time is low (≤ 5 s)—this is the result of unpredictable and non-uniform plasma distribution in the etch chamber when etch time is only 1 s. At higher etch times (30 s, 60 s, 100 s) the etch per second showed less variation and were closer to each other (45–50 Å/s) indicating good process control, repeatability, and reliability.

The resolution and repeatability of the process depends on our ability to precisely characterize the etch rate of Silicon, etch rate of SiO_2_ and the Si:SiO_2_ etch selectivity. Two characterization masks were constructed such that when lithographically aligned will contain small design patches of overlapping straight microchannels and square pillar arrays. Three different characterization wafers were etched for varying amounts with these two lithography masks on three different days. These two masks were etched for varying durations using a 600–800 W plasma of CHF_3_ and CH_4_ in 3:1 ratio in a reactive ion etching tool named Oxford RIE, to generate steps of step heights varying between 250 nm and 1.5 μm in the SiO_2_ layer. The SiO_2_ etching recipe was so chosen such that the SiO_2_:PR selectivity was ≥ 1. This ensures that the PR layer (4–10 μm) is always sufficiently thick to completely etch the thinner (3 μm) SiO_2_ underneath, thus eliminating one more parameter (SiO_2_:PR etch selectivity) from needing precise characterization. In this scenario, only the SiO_2_ etch rate information becomes important. The findings of SiO_2_ etch are summarized in Fig. 3. The raw data from which Fig. 3 has been constructed can be found in SI Table 2.

**Figure 4:**
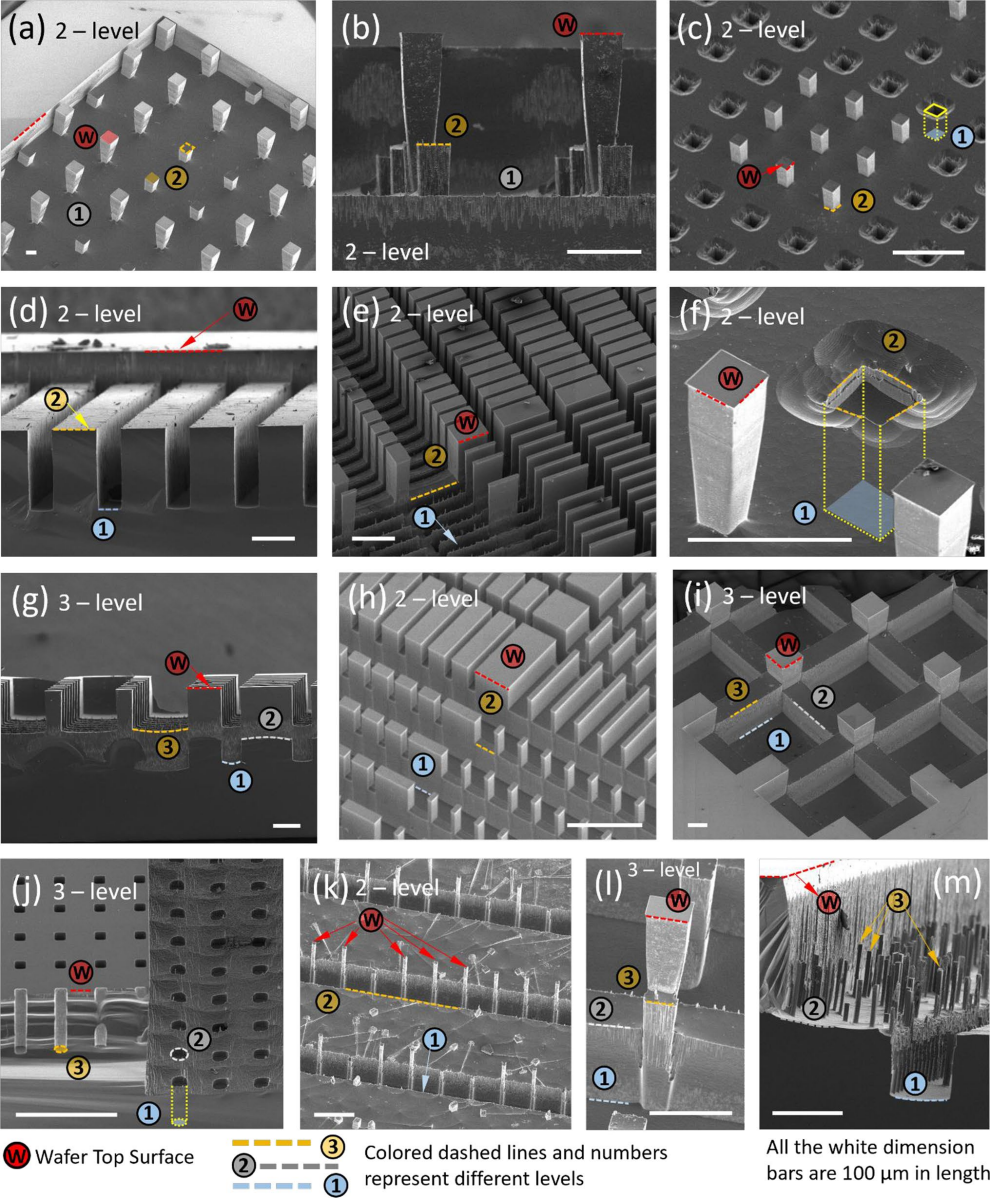
Demonstration of 3D structures using our fabrication method*. *(**a**) Multi-level pin fin structures (isometric view), these types of structures cannot be made via conventional chip stacking or double sided etching techniques; (**b**) (side view) multi-level pin fin array; (**c**) pin fins and pin holes; (**d**) straight microchannels offset from wafer surface (the ability to make microstructure slightly offset from surface has immense potential to ease bonding, integration and packaging different components together especially for extreme heat flux cooling applications); (**e**) serrated fin structures of different aspect ratios and spacing; (**f**) zoomed image of pin–fin pin hole sample; (**g**) (side view) Serrated Fin structure showing 4 distinct levels; (**h**) Isometric view of 2-level serrated fin; (**i**) Overlapping mask designs used to make 3-level serrated fins; (**j**) 3-level channels with pin-holes made by overlapping 2 mask designs. These kind of smaller pin–fin or pin-hole type structures distributed on a larger underlying meso structure is an easy and viable way to improve thermal performance of the active heat transfer zone in coolers; (**k**) Pin fins protruding from channel base taller than the channel sidewall height (some fins got broken during wafer dicing); (**l**) 3-level “Chair” design made by overlapping square pattern mask designs on the side of channels; (**m**) Initial concept of pin fin array patterned on two-level channels suggests to our ability to make well-ordered surface enhanced structures as well.

Following detailed characterization of the oxide etch rate using our specific recipe, we can precisely construct 3D structures in Silicon oxide. The target structures for our extreme heat flux cooling devices are extremely tall (~ 500 μm) needing 3–4 μm SiO_2_ layer as the mask. As mentioned before, we have chosen an aggressive oxide etch recipe with good SiO_2_:PR selectivity of ≥ 1. This is necessary in order to be able to etch the thick SiO_2_ layers (up to 4–6 μm) using a relatively thinner PR layer (4 μm, thus maintaining sub-10 μm resolution). Although, choosing an aggressive SiO_2_ etch recipe (with high etch per second value) leads to worsening of the vertical resolution of our target structures. As seen in Fig. 3, which plots the etch per second (Å/s) as a function of the total etch time and feature dimension, the average etch rate was well controlled within 45 and 54 Å/s for a wide range of target structures and for all etch durations above 1 s. At 1 s, the etching is severely starved of plasma and etch rate is much lower, ~ 30 Å/s. Additionally, in 1 s, plasma does not have enough time to distribute in the chamber uniformly thus also leading to Aspect Ratio Dependent Etching (ARDE), where the feature dimensions influence the etch rate more strongly (this can be seen in gray (1 s etch) line plot in Fig. 3). Thus, the vertical resolution of the 3D structures using our etching recipe is determined by a minimum of 2 s etch and is limited around 10 nm in the SiO_2_ layer. This translates to around 2–3 μm when the step is scaled and transferred to the Silicon wafer through DRIE. The etching recipe can be tuned (flow rates of respective gases can be reduced, CHF_3_ and CH_4_ ratio could be decreased) to make it less aggressive, and thus reduce the etch per second value—this will lead to better control of the etch, and better (sub-10 nm) resolution in the SiO_2_ 3D structure although at the cost of lowering SiO_2_:PR selectivity. Following characterization of the oxide etching step, a deep silicon etching recipe was used in the Plasma-Therm Deep Silicon Etcher (PTDSE) for pattern transfer. This recipe was also characterized using a test wafer with straight channels of widths 100–200 μm. The average Si:SiO_2_ etch selectivity over a total 200 μm depth of etch was found to be around 270–290. This etching recipe was developed extensively by a previous work by Hazra et al. who reported etch selectivity of 220–240, and etch rate of 8 μm/min^54^. The DSE recipe used by Hazra et al. was also extremely aggressive in order to accommodate their extreme total etch height of 1000 μm, although this aggressive recipe leads to a reduced Si:SiO_2_ selectivity. In our present study, the recipe was slightly modified (the silicon etching step, ‘etchA’ time was reduced to 3.1 s from 3.3^54^) to increase selectivity and attain straighter, more anisotropic etch profile. The progression of etch for our characterization structures (perpendicularly placed straight channels arrays of different widths and spacings between 100 and 400 μm) were investigated. 172 μm of Silicon was found to be etched for 0.61 μm of oxide, thus making the average Si:SiO_2_ etch selectivity ~ 282. Some of the final multi-level structures obtained through this process flow are shown in Fig. 4.

Finally, it is worthwhile to mention that the proposed fabrication concept, which involves multi-lithographically patterning a thin, low aspect ratio masking layer and then transferring that pattern to an underlying substrate through single-shot etching can be extended to other mask material and substrate combinations as well. Instead of CVD Silicon dioxide, thin metal layers (Au, Pt, Cr, W, Al) or other oxide (Alumina) or Nitride (SiN_x_) material can also be used as the mask layer. Superior etch selectivity of 10^5^ has been observed during DRIE of Silicon with an Al mask layer^31^—thus combining this with our method will enable the creation of extreme aspect ratio (≥ 35) multi-level structures. These new masking materials can also be deposited or grown on our wafer through other techniques like evaporation, sputtering, atomic layer deposition (ALD) or electroplating—thus making the concept applicable in wide range of fabrication scenarios. For different sets of mask and substrate material, the characterization process stays relatively unchanged, with a single run required using a characterization mask to quantify the etch rates and selectivities specific to the tools and process conditions used—these parameters are then to be used to design the process flow for obtaining our final target multi-level structures.

## Results and discussion

Different types of multi-level features made using this method, with varying feature widths and heights and topographies are presented in Fig. 4. All the structures in Fig. 4 are made via two rounds of lithography by overlapping two lithography masks. In theory, several lithography rounds can be performed on the wafer to create $n$-level structures.

**Figure 5:**
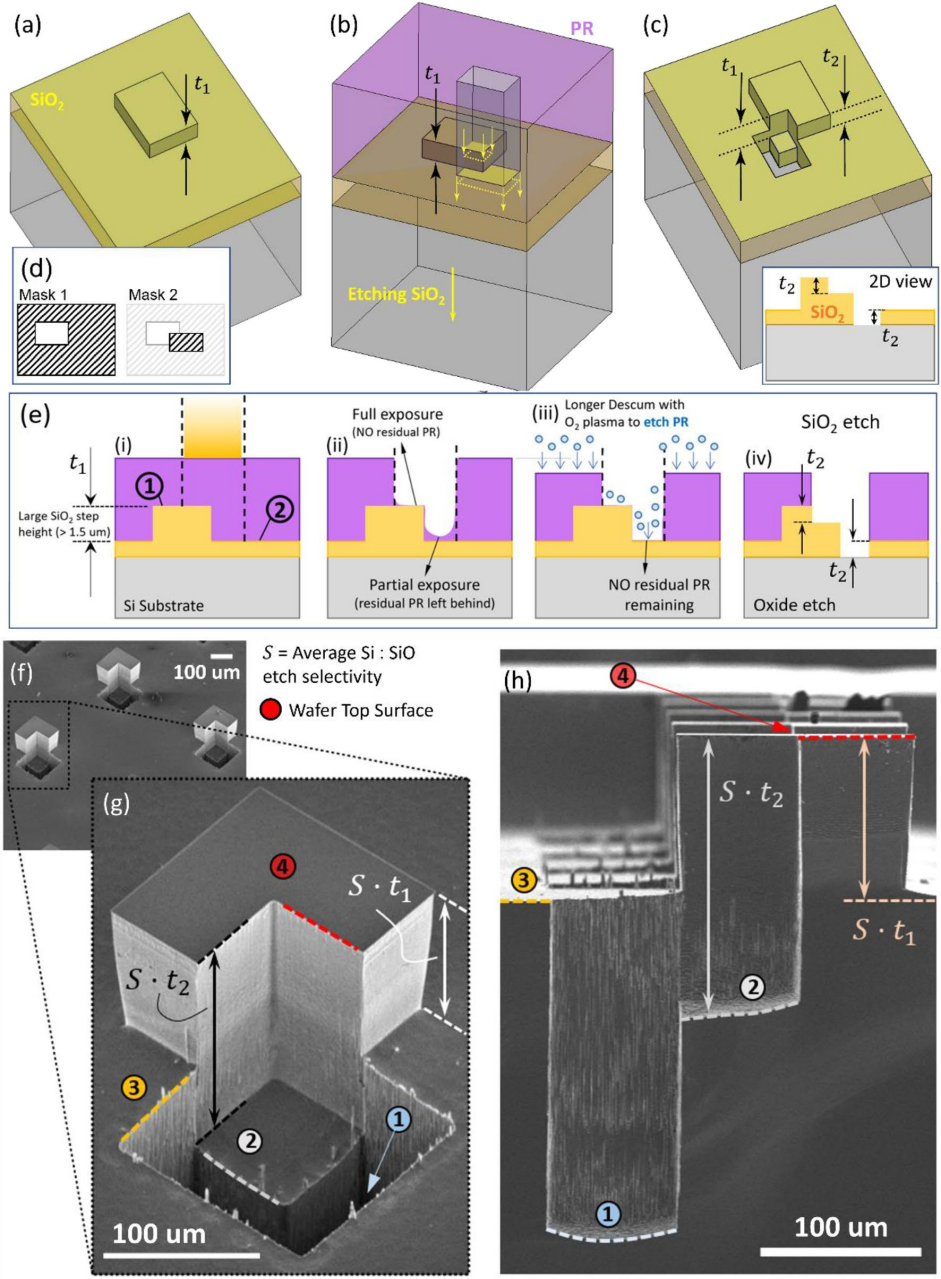
Overlapping mask designs to make complicated structures*.* (**a**) First mask design (as shown in (**d**)) is exposed and SiO_2_ is etched by ${t}_{1}$ amount; (**b**) Second design overlaps partially on the step made in step (**a**) and (**c**) SiO_2_ etch is performed to obtain a 3-level 3D feature on the SiO_2_. A 2D counterpart of this 3-levels structure is shown in (**e**—iv) (**d**) The two masks for two rounds of ‘litho + SiO_2_ etch’ are shown, image on the right shows how the masks overlap. (**e**) It has been verified that steps up to 1 μm in SiO_2_ do not affect the exposure process. In case the step height is ≥ 1.5 μm, there could arise differences in exposure quality between the two levels of SiO_2_, 1 and 2. In this situation the lower steps might be underexposed, with PR being left behind—a longer downstream descumming step (≥ 2 min) fixes the issue, by removing all of this residual PR. (**f**) After DRIE, the structure is scaled vertically and transferred to the underlying Si, and the new 3-level pin–fin–hole combination arrays are shown; (**g**) zoomed view of the 3D structure; (**h**) side cross-sectional view. The numbers represent the different levels.

Usually, the number of ‘lithography + SiO_2_ etch’ steps is equal to the number of levels required in the multi-level structure (observe Fig. 2a–i), although it was soon realized that further simplifications could be made easily to reduce the number of processing steps required for these structures. For example, the final step height in the SiO_2_ layer could be entirely replaced with a baked Photopolymer, thus reducing one round of ‘litho + SiO_2_ etch’. Although this would require designing the fabrication flow while accounting for the different etch rates and selectivities of PR, SiO_2_ and Si during the process. In addition to these simplifications, mask designs themselves can be cleverly combined and overlapped between different rounds of ‘litho + SiO_2_ etch’ which gives rise to more levels using lesser number of rounds of ‘litho + etch’. An example has been demonstrated in Fig. 5, where two rounds of ‘litho + etch’ involving 2 masks could generate a 3-level structure. More such structures are seen in Fig. 4g,i,j,l,m all of which are made by overlapping 2 masks (the exact mask designs are left as exercises for the reader).

Figure 5 shows a multi-height pin fin structure with a pillar and pocket feature etched together. It also briefly discusses an issue that might arise during the exposure phase of lithography while making extremely tall structures. The dimensional accuracy and exposure quality by the MLA Heidelberg Maskless aligner (exposure tool) depends on two main parameters—the exposing light energy (dose) and the location of focus (defocus) with respect to the PR top surface, although the exposing energy is the primary determinant. It has been observed previously that a ± 1 μm change in the defocus value from the optimal focal plane does not affect the exposure step significantly—this suggests that if the 3D features and step heights in the SiO_2_ is low enough (sub-micron), exposure quality at the two steps is relatively good. Overlapping designs were tested for step heights from 0.5 to 1 μm and exposures were found to be satisfactory on both the levels (Fig. 5a–c). When photolithography is attempted on SiO_2_ step heights which are more than 1.5 μm apart, the exposure qualities on the two different levels of the SiO_2_ might be slightly different (as shown in the schematic in Fig. 5d–e, one of the levels might be over or underexposed) and this should be taken into consideration during the exposure step. One easy and quick fix is to choose the focal plane of exposing light such that full exposure lithography happens at the upper level or the top of the step. This would simultaneously mean that the lower level (bottom of the step) is under-exposed and residual PR might be left behind. This can be taken care of by increasing the downstream descumming step duration to longer durations (≥ few mins). The extra low power (50 W) O_2_ plasma descumming time would remove all the residual PR from the bottom surface of the SiO_2_ step and solve this issue. Although, these cases might benefit from a slightly thicker (≥ 5 μm) initial PR coat to accommodate for the extra PR being etched during the descumming step—this will ensure there is still sufficient PR left to completely etch the underlying SiO_2_ layer even after the longer descum step.

### Impact

The novel approach delineated in this letter allows us to precisely create multi-level, hybrid structures through an easy to characterize and standardizable process flow. Some of these kinds of structures are demonstrated in Figs. 4 and 5, but the possibilities are endless. Since most of these structures are tall (≥ 100 μm, often as tall as 500 μm) with high (5–10) to extreme (15–25) aspect ratios, we anticipate these kinds of structures to be best suited for micro to meso-scale microfluidics and liquid cooling applications. The ability to create 3D, multifunctional and hierarchical structures is especially important to the academic and industrial research environment right now, since myriads of micro-meso scale applications can benefit in performance by merely replacing conventional single-level device structures into multi-functional and multi-level, hybrid features. Some of those applications are listed in the following section.

### Improvements in microfluidics

Flow type microfluidic devices have active regions with diverse range of functionalities, some examples being mixing, particle separation, sorting, separation, and analysis^5,32–36^. Alongside the active region, the devices also consist of flow channels, inlets and the outlets which are usually of different feature sizes and at different levels in the device—flow channels are wider, inlets and outlets through etched to enable flow connections in and out of the device. One of the most common approaches for high volume manufacturing of such devices is thermal or UV Nano Imprint Lithography (NIL)^14,15^. This utilizes a rigid master or mold (often made from Si) which is used to create the mirror inverse out of several soft polymers like SU-8, PDMS, Polyurethanes (PU), Polycarbonates (PC), PMMA etc. Currently, no method exists for creation of multi-level rigid molds^18^—our method will be immensely useful in this context. Additionally, this process will enable easy fabrication of active area microstructure and flow channels with independent control of the feature widths and heights, which will pave the way for multi-physics on the same device or chip^57,58^. Complicated flow paths and internal capped structures like the ones demonstrated by Duong et al.^59^ by 3D-printing can now be made by ease by bonding two silicon chips or their corresponding NIL casted polymers. Digital or droplet-based microfluidics could also immensely benefit from such multi-level structures^57^. Carefully crafted multi-level pins and holes (like the ones shown in Fig. 4c,f) combined with multi-level channels could be used to create, trap, and transport droplets effectively. Recently, hybrid structures have garnered a lot of attention from the optofluidic community as well—Parks et al. demonstrated the integration of a PDMS based fluid handling layer with a silicon optical sensor for single particle detection but also showed its functionality for other purposes like labelling DNA, single molecule detection, particle mixing, distribution, and filtering^60^. Another benefit to multi-level featuring is the ability to make surface structures will also present several possibilities in designing bio-inspired surface designs with targeted functionalities^13,39^, for example, superhydrophobic, self-cleaning lotus leaf, antifouling and drag reducing shark skin and mollusk shell textures, anti-reflective moth-eye, photonic butterfly wing structures and “water harvesting” micro-bumps like Namib beetle skin. Being able to combine several different functionalities together on the same chip will push us to create more versatile lab-on-a-chip (LOC) devices^18^ which will have a massive impact on bio-microfluidics^5,58,60–62^, enabling droplet based small volume sample-reagent testing, biological and chemical assays, point-of-care diagnostics, cell and DNA manipulation^5,61,62^ and testing, separation^35^, sorting^34^, and analysis^36^. These types of multi-level materials will also have varied use in situations requiring surface and absorption enhancements, some of which are water absorption, desalination, carbon capture, battery technology, adsorption enhancement, catalysis, surface tension or capillary force driven transport^6–12^ etc.

### Advances in convective cooling devices

Moreover, hybrid multi-level structures probably have the most significant impact on improving the device performance in the field of embedded liquid cooling solution. Hybridization of the Cold Plate side microchannel (by introducing a microwick or surface features at the bottom of a straight microchannel) lead to increased thermal performance in forced fed microchannel cooling scenarios^8^. Zhu et al. reported heat transfer coefficient improvements from 17% to over 117% for microstructured microchannel compared to smooth microchannel, for 25 and 75 µm tall micropillars, respectively, using methanol as the working fluid without significant increase in pressure drop^56^. Passive heat spreaders like Heat Pipes and Vapor chambers with hybrid, bi-porous wicks instead of a conventional mono-porous one showed significant improvement in their heat spreading capabilities^40^. Dai et al. demonstrated that a complex hybrid wick when used in a heat pipe, leads to a massive 30 folds increase in maximum spreadable heat load as compared to solid Copper^41^. Zhou et al. validated the superior performance of hybrid two-level wicks in Vapor Chambers by reporting a 28% and 17% decrease respectively in device thermal resistance as compared to a state-of-the-art commercial monoporous and biporous wick TGP (Thermal Ground Plane)^42^. Moreover, our ability to reliably create multi-level hierarchical structures will allow us to aggressively scale up forced convection based active cooling device using a second 3D manifold layer for efficient fluid delivery. High performance cooler scale-up is an immensely important goal being pursued in the field of embedded cooling; this will allow us to pack energy dense power electronic components closely together and continue the trend of improving electronics speed and energy density^8,43,52,54^. Pan et al. performed numerical simulations in ANSYS Fluent to compare Manifolded Coolers (MMC) design with Traditional 2D Coolers (TMC)s and showed that at same flow rates, the MMCs can achieve similar levels of thermal performance as the TMCs but achieve a massive 4× to 6× reduction in total device pressure and thus, 4× to 6× improvement in Coefficient of Performance (COP)^43^. In addition to active coolers, such hybrid, multi-height wicks will also enable scale up of heat spreader technologies. This is possible since multi-depth features when cleverly combined in the evaporator wick can effectively solve the mass transport limitations inherently imposed by thin evaporator wicks in liquid-to-vapor phase change heat spreaders^8^. In ultra-thin vapor chamber designs, the short pillars could be placed over the hot spots to hold a very thin liquid film, leading to smaller thermal resistance and superior thermal performance while the tall pillars will act as liquid replenishment routes supplying enough wicking mass flow from the condenser back to the evaporator over large device areas. In addition to the above mentioned uses for multi-level structures, the active heat transfer 3D micro-featured surfaces themselves can be surface enhanced (Fig. 4m shows surface enhancements on top of 2-level structured channels to make an overall 3-level structure) using this novel method. These surface enhancements will be well-ordered, and their dimensions exactly controlled, thus they can replace conventional methods of creating stochastic surface roughness elements (wires^44^, tubes^38^, needles^63^, broccoli^64^, polyp^65^) which are harder to control and repeat. These surface enhancements lead to massive improvements in device metrics by enhancing the capillary wicking-based transport from the condenser back to the evaporator. This has been demonstrated by creating hybrid wicks using UV laser induced roughness^64,65^, hydrothermal ZnO Nanowire synthesis on silicon microstructure^56^ and then performing capillary rate of rise tests to show that their wicking rate is much faster than their non-hybrid smooth counterparts with no surface enhancements. Surface enhancements combined with multi-level structures, additionally increase the overall surface area available for heat transfer in forced fed convection and thin film evaporation scenarios^66^, and leads to increased bubble nucleation sites in pool and flow boiling regimes^7,8,44^.

Some of the above-mentioned applications help motivate the superiority of hybrid structures. Several orders of improvement will be achieved in many applications when conventional monoporous or single-level wicks are replaced by hybrid, multi-level wicks. We hope that this standardized recipe for manufacturing such multi-level structures will encourage more research, and eventually adoption of such structures in commercial devices and real-life scenarios.

## Conclusion

In this letter, we have detailed a novel Silicon Oxide (SiO_2_) to Si pattern transfer process which uses multiple lithography to first pattern a thin, low aspect ratio SiO_2_ layer which is then transferred to the Si substrate underneath via a one-shot deep etching technique. The extreme high etch selectivity between Si:SiO_2_ etch of 200–300 enables us to create multi-level structures of extreme heights of ≥ 500 μm and extreme aspect ratio (≥ 10–15) in Silicon with a relatively thin (≤ 3) oxide layer on top. With the current oxide etch recipe selected, we obtain a stable resolution of 90–100 Å in SiO_2_ for 2 s of etch, this yields a vertical Si etch resolution of 2–3 μm in Si. With more tweaks to the etch recipe, the oxide etch per second can be reduced and resolution further refined. This process provides several advantages over conventional chip stacking and grayscale lithography approaches, which have been summarized in detail in Supplementary Information Table 3. The novel process has been used to create myriads of multi-level structures as shown in Fig. 4, furthermore, Fig. 5 shows how multiple lithography masks can be overlapped to create more levels using lesser number of lithography steps. Such multi-level structures in the micro and mesoscale have far reaching applications in the fields of microfluidics, cooling technologies, biology, filtration, and energy as mentioned briefly in the Introduction and Impact sections. Furthermore, our novel process solves several practical characterization and standardization challenges that mar the use of grayscale lithography and conventional LELE process, to make multi-level hierarchical structures and thus arguably is more suited for commercial mass manufacturing, high throughput situations. Difficult, non-standard fabrication with tight process tolerances is the primary reason why multi-level, 3D structures are not regularly observed in commercial devices. Having a standard recipe that can be easily translated from one lab to another will open a range of possibilities in research and development of such hybrid structures for the improving functionalities and increasing their performance by many folds.

## Supplementary Information

Supplementary Information.

## Data Availability

All data generated or analyzed during this study are included in this published article and its Supplementary Information files.
