# Supplementary material for: A novel hardmask-to-substrate pattern transfer method for creating 3D, multi-level, hierarchical, high aspect-ratio structures for applications in microfluidics and cooling technologies
Source: Sci Rep. 2022 Jul 16;12:12180. doi: 10.1038/s41598-022-16281-5 (PMC9288478; doi:10.1038/s41598-022-16281-5)
Supplement: Supplementary file 1 — Supplementary Information. [file 41598_2022_16281_MOESM1_ESM.docx]

**A Novel Hardmask-to-Substrate Pattern Transfer Method for Creating 3D, Multi-level, Hierarchical, High aspect-ratio Structures for Applications in Microfluidics and Cooling technologies**

Sougata Hazra*, Chi Zhang, Qianying Wu, Mehdi Asheghi, Kenneth Goodson, Ercan M. Dede, James Palko, Sreekant Narumanchi

* Corresponding author, email: [shazra@stanford.edu](mailto:shazra@stanford.edu)

**Supplementary Information**

As mentioned earlier, the fabrication process flow involves very commonly used tools and processes – SiO_2_ deposition, photolithography, SiO_2_ etch and Deep Si etch. The specific process flow, tools and process condition that was followed in this letter is detailed in Table 1.

Repeat $n$ times for $n$ levels

| **Table 1: Process flow for creating n-level 3D structure in Si wafers** | | | | |
| --- | --- | --- | --- | --- |
| # | | Tool | Function | Process Condition |
| 0 | | Wet bench | Clean wafer | The wafer is first cleaned in hot (120^0^C) piranha soln. (75% H_2_SO_4_, 25% H_2_O_2_), rinsed and dried thoroughly. |
| 1 | | CCP dep | Plasma Enhanced CVD system | N_2_O gas flow (with small amt. of SiH_4_, He) at 350^0^C was used in the process chamber at 200W Rf power to CVD deposit SiO_2_ on the wafer surface. SiO_2_ can also be deposited at lower temperatures of 90^0^ – 120^0^C with high density plasma enhanced CVD. Higher quality, defect-free SiO_2_ can also be grown on the Si wafer directly using dry/wet oxidation in a high temperature (> 800^0^C) ovens. |
|  | 2 | SVG coat | Coat Photoresist | The wafer was spun at 1500 RPM for 30 seconds while Shipley 220–3 PR was puddle dispensed on the center of the spinning wafer. This yields a uniform 4 um thick coat of PR. It is important to ensure that the PR layer is thicker than the features etched in the SiO_2_ underneath, in order to overcome any challenges associated with spin coating. But simultaneously, the PR thickness should not exceed 7 – 10 um in order to ensure good dimensional accuracy of sub 10-micron features after exposure and development. |
|  | 3 | MLA 150 Heidelberg Inc. | Expose design | Maskless aligning tool using 405 nm UV light to expose Shipley resists. Characterization was first performed on a wafer to determine the optimal exposure energy (260 – 280 mJ/cm^2^) and focus level (2 to 3 um above the PR surface) for 4 um thin coat of SPR220-3 and was used to expose the design. |
|  | 4 | Wet bench | Development | The wafer was developed through three rounds of (1 min dip in MF26A + 10s rinsing in water) after which it was spun dry at 6000 RPM |
|  | 5 | Technics Asher | Descum | Low energy (50W) O_2_ plasma at 27 sccm flow rate was used for 30 – 45 sec to remove residual PR after development |
|  | 6 | UV cure | The wafer was placed under UV light for 10 – 15 mins to harden the remaining resist | |
|  | 7 | Oxford RIE | Oxide etch | ICP Plasma etch was performed with CHF_3_ (45 sccm) and CH_4_ (15 sccm) at 600 – 800W power at 100 mTorr chamber pressure. Etch selectivity with PR was found to be 2.5 and the etch rate of SiO_2_ was precisely measured as a function of exposing design. (Table 2) This information is obtained from the first characterization run, and then used later to design the final structures. |
|  | 8 | Matrix | Strip PR | High power O_2_ plasma was used to strip the PR. It is important that this step be dry – very thin sections of the SiO_2_ layer was observed to detach from the Si wafer and migrate to other parts when wet acetone sonication was used to strip the PR. |
| 9 | | PTDSE | Deep Si Etch | A Bosch time-multiplexed process using SF_6_ and CF_4_ plasma was used to etch the 3D pattern of the oxide layer into the Silicon. The etching recipe used has been slightly modified from one detailed in [32] |

Table 1: Specific tools used for our process flow. These steps have been performed at the Stanford Nanofabrication Facility

A characterization mask consisting of straight channels and square pillar arrays has been used to estimate the etch per second of SiO_2_ and the feature loading dependent etch rate. Table 2 shows the etch progression in the characterization mask. The characterization mask consists of square pillar arrays and channels of varying dimension. This Table has been used to construct the plots in fig. 3.

Table 2: Characterization data using pillar arrays and straight channels for our oxide etch step using Oxford RIE

| **Table 2: Characterization data of SiO_2_ etch using Oxford RIE** | | | | | | | | | | | | |
| --- | --- | --- | --- | --- | --- | --- | --- | --- | --- | --- | --- | --- |
| Wafer 1 | | | Cycle 1 (120 sec) | | C 2 (120 s) | | C 3 (120 s) | | Cycle 4 (0) | | Cycle 5 (0) | |
| Feature type | Dimension | Initial SiO_2_ thickness | Amt. etch (dh) | Etch / sec (eps) | dh | eps | dh | eps | dh | eps | dh | eps |
|  | (um) | (nm) | (nm) | (nm/s) |  |  |  |  |  |  |  |  |
| Ch | 200 | 1560.3 | 479.6 | 39.97 | 470 | 39.2 | 462.1 | 38.51 | - | - | - | - |
| Ch | 300 | 1560.5 | 482.7 | 40.22 | 470.1 | 39.2 | 490.9 | 40.91 | - | - | - | - |
| Ch | 400 | 1560.3 | 495.2 | 41.27 | 470.2 | 39.2 | 480.9 | 40.01 | - | - | - | - |
|  |  |  |  |  |  |  |  |  |  |  |  |  |
| Wafer 2 | | | Cycle 1 (30 sec) | | C2 (5 sec) | | C3 (1 sec) | | C4 (60 sec) | | C5 (100 sec) | |
| Ch | 120 | 11491 | 1368 | 45.6 | 255 | 51 | 34 | 34 | 2849 | 47.48 | 4568 | 45.68 |
| Ch | 120 | 11553 | 1428 | 47.6 | 272 | 54.4 | 31 | 31 | 2947 | 49.12 | 4760 | 47.6 |
| Ch | 150 | 11538 | 1442 | 48.07 | 290 | 58 | 32 | 32 | 3017 | 50.28 | 4778 | 47.78 |
| Ch | 180 | 11459 | 1351 | 45.03 | 260 | 52 | 32 | 32 | 2855 | 47.58 | 4554 | 45.54 |
| Ch | 250 | 11519 | 1452 | 48.4 | 279 | 55.8 | 32 | 32 | 3020 | 50.33 | 4829 | 48.29 |
| Ch | 300 | 11547 | 1411 | 47.03 | 276 | 55.2 | 33 | 33 | 2955 | 49.25 | 4732 | 47.32 |
| Sq. Pi. | 10 / 30 | 11505 | 1431 | 47.7 | 252 | 50.4 | 46 | 46 | 2954 | 49.23 | 4714 | 47.14 |
| Sq. Pi. | 20 / 60 | 11503 | 1413 | 47.1 | 269 | 53.8 | 52 | 52 | 2920 | 48.67 | 1571 | 45.71 |
| Sq. Pi. | 50 / 110 | 11465 | 1353 | 45.1 | 289 | 57.8 | 35 | 35 | 2888 | 48.13 | 4630 | 46.3 |
| Sq. Pi. | 40 / 120 | 11485 | 1395 | 46.5 | 252 | 50.4 | 53 | 53 | 2927 | 48.78 | 4715 | 47.15 |
| Sq. Pi. | 50 / 150 | 11483 | 1360 | 45.33 | 290 | 58 | 34 | 34 | 2917 | 48.62 | 4668 | 46.68 |
| Sq. Pi. | 60 / 180 | 11509 | 1426 | 47.53 | 267 | 53.4 | 26 | 26 | 2942 | 49.03 | 4688 | 46.88 |
| Sq. Pi. | 70 / 250 | 11475 | 1385 | 46.17 | 275 | 55 | 28 | 28 | 2915 | 48.58 | 4685 | 46.85 |
| Sq. Pi. | 100 / 300 | 11449 | 1377 | 45.9 | 266 | 53.2 | 25 | 25 | 2908 | 48.47 | 4632 | 46.32 |
| Sq. Pi. | 150 / 450 | 11507 | 1415 | 47.17 | 272 | 54.4 | 31 | 31 | 2947 | 49.12 | 4711 | 47.11 |
| - Note: Ch = Straight Channels; Sq. Pi. = Square Pillar Array; Dimension – width (Channels) and diameter / pitch (Sq. Pi.) | | | | | | | | | | | | |

| The following is a tabular comparison between our novel process flow with existing methods for creation of hybrid multi-level structures. | | | | |
| --- | --- | --- | --- | --- |
| **Table 3: Detailed comparison between existing methods and our novel hardmask-to-substrate pattern transfer process flow** | | | | |
|  | 1. Chip Stacking | 2. Grayscale Lithography (Photoresist – Si substrate) | | 4. Novel SiO_2_-Si pattern transfer (PR – SiO_2_ – Si) |
|  |  | A. Optical Mask Assisted | B. Maskless Direct Write |  |
| Brief Description | Produces multi-level 3D structures by breaking them in multiple 2.5D structures – current commercial go-to  (fig. 1 (f), (g), (h), (i)) | Relies on accurately designed micro-nano scale patterns in the gray zone of an optical photomask, which partially blocks the full exposure dose to achieve a gray dose and partial development of the PR. 3D pattern on the PR is transferred to Si through DRIE etch. | Instead of an optical photomask, this process depends on the exposure tool’s capability to directly modulate exposure energy between different parts of the design to achieve varying amounts of PR washed away from these zones during development. | Multiple rounds of (maskless direct write full exposure photolithography + SiO_2_ etch) is performed to make a 3D pattern on a thin SiO_2_ layer on top of the Si. Compared to grayscale lithography, an additional SiO_2_ layer is introduced between the PR and Si substrate, which will now act as the etch stop layer during DRIE etch. |
| Lithography Mask | Optional. Both masked methods or maskless methods can be used for full exposure lithography. | YES, requires a physical optical photomask   - Very expensive to fabricate [27, 37-42] - Designing step requires multi-objective complex modelling to determine gray zone pattern dimensions – tedious [22] - Designing mistakes are costly – leads to wastage of processing time and money | NO, does not require a physical mask   - Digital mask increased room for error during design - No wastage of time and money associated with physical Chrome-on-glass mask fabrication | NO, does not require a physical mask  Although, our process involves full exposure lithography only, thus maskless litho can be replaced by multiple masked lithography for mass manufacturing scenarios (note, this is easy since no gray litho masks are involved) |
| Etch stop layer | Usually PR | Photoresist (can be up to 5μm thick) | Photoresist (thin, usually < 2μm) | Ultra-thin (up to 3 – 4μm) SiO_2_ layer |
| Etch Selectivity | Up to 100 | Up to 100 for specific design and RIE recipe | Demonstrated up to 30 | 200 – 280 |
| Total feature height demonstrated | Wafers in the middle of the stack can NOT have free standing structures. Eg. A micro-pillar array with different heights cannot be made using this method since pillars are free standing features. | Up to 250μm [24] | - Low height, 10μm based on more than 10 published research articles [27, 37-42] - Eckstein et al. [40] developed a special illumination tool to achieve 75μm tall structures - Heidelberg instruments, a premier maskless tool manufacturer reports an upper height limit of 60μm [22, 40] | - No restrictions on structure type like in Chip stacking or double sided processing. - Capability more than 500 – 600μm (demonstrated up to 350μm)   The use of high etch selectivity SiO_2_ as the intermediate layer solves the issue of low feature heights. Using 3μm of SiO_2_ potentially enables us to create > 600μm tall structures. |
| Feature lateral dimensions | Sub-10μm resolution | ~ 10μm or much more than the pixel dimension (1 – 2μm) in gray zones. | Sub 10-μm resolution | Sub-10μm resolution |
| Number of gray-levels  (Maximum number of levels in the structure that can be made in the multi-level structure) | $n$ gray levels require $n$ wafers carefully ground to specified level heights | Limited by the mask fabrication capability, stepper resolution, upper dose level without pixel recreation | Limited by stepper resolution, PR response curve | No limit (number of lithography step which can be as few or as many = number of levels)  Masks can be intelligently overlapped to reduce number of lithography steps |
| Etch stop layer profile control |  | Most issues regarding etch stop profile arises during the gray level exposure step | | |
|  | Standard | Very difficult even with a perfectly difficult mask [22, 26]   - Rough PR surface after partial development - Gray zone profile tapering, “well”-ing - Higher dose unwanted pattern recreation | Very difficult [27]   - Gray zone profile tapering, “well”-ing - messy PR surface after partial exposure and development - High post etch surface roughness | Standard – Our process eliminates all gray exposure steps. Full exposure multiple round photolithography is used to 3D pattern the SiO_2_ layer with the steps being perfectly vertical anisotropic (SiO_2_ wall profile angle can be changed too by tuning oxide etch recipe) |
| Etch stop profile correction | Standard | Difficult – Expensive (physical mask) and time-consuming (mask making lead time) | Two methods which are both expensive and cumbersome [22] –   - Through thorough characterization – tedious - 3D proximity effect correction (PEC) – expensive | Profile correction only involves characterizing oxide etch rate as a function of feature dimension which can be easily done using a simple characterization mask |
| Characterization Step | Difficulty of the etch stop layer profile control and correction directly correlates with the difficulty of the characterization | | | |
|  | Only etch needs to be characterized  Extremely low yield (50 – 60%) since it requires handling of fragile wafers with deep etches [32, 33] | Extremely difficult characterization step [27, 37–42] –   - Difficult profile control and correction - Each design requires extensive, individual, characterization - involves making a detailed dependence map from pattern dimension 🡪 reduced light intensity 🡪 PR height – this is difficult and cumbersome especially with physical masks - Dimensional inaccuracies by gray zone profile distortion | Extremely difficult characterization step [22, 27, 37–42] –   - Difficult profile control and correction - Each design requires extensive, individual, characterization - Feature dimension dependent PR response curve shifting - Dimensional inaccuracies in gray exposure regions - Almost impossible to correct profile distortion issues (e.g. Profile “well”-ing) that arise due to gray level exposure | Standard, only etching needs to be characterized –   - Easy profile control and correction - Individual Characterization step NOT required for each design - Full exposure photolithography eliminates all the issues regarding profile distortion and dimensional inaccuracies - For each lab (set of tools), one etching run using a characterization mask is sufficient to quantify Si, SiO_2_ etch rates and selectivities. These data can then to be used to design masks for the final target structure. |
| Standardizability | High level of failure at bond sites during stress cycling [35, 36] | Difficult because of gray profile distortion issues | Difficult because of gray profile distortion issues | Easy (no gray exposure step). Single etch step replaces multiple ones thus eliminating any manual handling. Also, since the multi-level structure is made of one wafer no bonding required |
| Process tolerances |  | Tight (narrow window of process parameters) | Tight (narrow window of process parameters) | No tight process tolerance (easy to use tools and processes) |
| Knowledge transfer | Easy | Difficult because of tight process tolerance | Difficult because of tight process tolerance | Easy, because of relatively simpler characterization and standardization steps. |
| Potential for commercial use | Commercially used | Difficult to use commercial because of poor process reliability, difficult characterization, tight process tolerance [27, 37-42] | Difficult to use commercial because of poor process reliability, difficult characterization, tight process tolerance [22] | Easy since it is easy to characterize and standardize. This recipe also uses very commonly used tools and processes (SiO_2_ CVD deposition, lithography, SiO_2_ and Si etch) so easily integrable with existing cleanroom-based manufacturing lines |
| Process cost | $$$ (expensive, wafer thinning, bonding, increased cost due to high failure rate) | $$$$ (most of it coming from physical masks and the characterization required to design such masks) | $ (process)  $$ (profile correction, characterization, and distortion correction) | $$ (slightly additional cost compared to 3B, associated with multiple rounds of lithography and SiO_2_ etch) |

Table 3: Detailed comparison between existing methods and our novel hardmask-to-substrate pattern transfer process flow
